# A Comprehensive Review on Changes in Rhizosphere Soil Mediated by Microplastics: Soil Property, Microbial Gene Expression and Crop Growth

**DOI:** 10.3390/microorganisms14091879

**Published:** 2026-08-24

**Authors:** Xin Jiang, Xianfei Huang, Xianliang Wu

**Affiliations:** 1 Guizhou Key Laboratory of Plateau Wetland Conservation and Restoration, Guizhou Normal University, Guiyang 550025, China; jiangxin@gznu.edu.cn; 2 Guizhou Institute of Biology, Guizhou Academy of Sciences, Guiyang 550009, China; wuxianliang1995@163.com

**Keywords:** microplastics, rhizosphere soil, response mechanism, enzymes, viruses

## Abstract

Microplastics (MPs) pollution caused by agricultural film residues, organic fertilizer application, sewage irrigation, and atmospheric deposition has gradually become an unignorable interference factor to the sustainable development of the rhizosphere soil and crop in farmland. However, their specific impacts on the rhizosphere and crops remain unclear. Therefore, this review focuses on the current knowledge on the response mechanisms of rhizosphere soil and crops to MP contamination. The density of MPs is generally lower than that of soil mineral particles. Their substantial accumulation in soil can significantly reduce both the bulk density (by increasing total porosity) and the particle density (by diluting the heavy solid phase with light plastic components). The introduction of MPs disrupts the normal metabolism of soil bacterial communities; a disruption directly reflected in functional genes associated with carbon cycling. MPs can interfere with the activity of key metabolic enzymes involved in fungal nutrient cycling, thereby disrupting normal energy allocation and material metabolism. Viruses can regulate the turnover and metabolism of microbial communities through lytic and lysogenic cycles, consequently influencing the carbon fate of MPs. The toxicity and underlying mechanisms of MPs on soil fauna primarily manifest in aspects such as feeding behavior, growth and development, oxidative stress, intestinal toxicity, and reproductive toxicity. The direct effects of MPs on plants include physical barriers and mechanical damage, induction of oxidative stress, interference with nutrient uptake, disruption of photosynthesis and carbon metabolism, and disruption of plant hormone networks. This review identifies critical knowledge gaps, particularly regarding crop quality, field-based soil faunal studies, virus-microbe interactions, and degradation products, and proposes future research directions to better understand the risks MPs pose to agricultural sustainability and food safety.

## 1. Introduction

Microplastics (MPs), as an emerging class of pollutants, are generally defined as plastic particles smaller than 5 mm and have attracted considerable research interest in recent years. Based on their origins, MPs are categorized into primary and secondary types [1]. Primary MPs are originally manufactured at microscopic sizes, such as plastic microbeads in cosmetics, synthetic textile fibers, and tire wear particles. Secondary MPs are formed through the physical, chemical, or biological fragmentation of larger plastic waste that has entered the environment [2]. MPs originate from diverse sources, including industrial emissions, agricultural film degradation, textile washing, and tire abrasion [3]. Once released, these particles migrate across various environmental media—via atmospheric deposition and surface runoff—and can even undergo global circulation through rainwater. They have been detected in soil, freshwater, oceans, and even polar regions. MPs are readily ingested by organisms, potentially causing digestive tract blockages or false satiety, which may lead to physical injury and malnutrition [4]. Degradable MPs are more prone to form nano-sized particles in soil; they can penetrate soybean root systems, resulting in an 18% decrease in chlorophyll content and a 22% reduction in aboveground biomass, while also affecting the activities of enzymes involved in soil carbon, nitrogen, and phosphorus cycling [5]. Owing to their high specific surface area, MPs can adsorb toxic substances such as heavy metals and pesticides. These adsorbed contaminants may be transferred and accumulated along the food chain, leading to combined pollution and amplified toxic effects. Human exposure to MPs occurs mainly through diet (food and drinking water), inhalation (airborne fibers), and dermal contact [6]. MPs can accumulate in human tissues, including blood, lungs, liver, and even thrombi, potentially triggering inflammatory responses and oxidative stress, and posing risks of cytotoxicity and neurotoxicity [7]. Therefore, it is urgent to investigate the mechanisms underlying MP-induced risks to human health, particularly from the perspective of their migration through rhizosphere soil, soil fauna, and plants.

In farmland, MPs primarily originate from agricultural film residues, organic fertilizer application, sewage irrigation, and atmospheric deposition, and have thus become a significant stressor in soil–plant systems [8]. Currently, research focus has gradually shifted from the environmental occurrence of MPs to their migration and transformation behaviors within these systems [9]. In particular, the interfacial processes and accumulation mechanisms of MPs in the crop rhizosphere microzone have emerged as a cutting-edge scientific issue at the intersection of environmental soil science and plant physiology [10]. The accumulation of MPs in the rhizosphere is a complex interfacial process involving physical, chemical, and biological mechanisms [5]. As a key interface and dynamic hotspot for soil–plant interactions, the rhizosphere possesses unique physicochemical properties and active microbial activities that profoundly influence the environmental fate of MPs [11]. First, MPs can enter root xylem through physical pathways—such as cracks or intercellular spaces at the edges of newly formed lateral roots—driven by transpirational pull, and can be further translocated upward, posing a direct risk of food chain exposure [12]. Moreover, MPs with different particle sizes (especially at the nanoscale), polymer types, and surface aging characteristics can significantly alter the aggregation structure, hydraulic properties, and nutrient cycling of rhizosphere soil [5]. By influencing the structure and functional activity of soil microbial communities, they indirectly regulate their own bioavailability. Importantly, MPs can act as carriers for toxic substances such as heavy metals and persistent organic pollutants, thereby forming combined contamination and significantly modifying the environmental behavior and biological toxicity of these pollutants through synergistic or antagonistic effects [1]. This further increases the complexity of their impacts on crop growth and the associated food safety risk assessments. Therefore, in-depth investigation of the multi-process accumulation mechanisms of MPs at the root–soil interface and elucidation of their interactions with the rhizosphere microbiome constitute the theoretical basis for accurately predicting their long-term ecological effects and for developing effective pollution prevention and control strategies.

**Figure 1:**
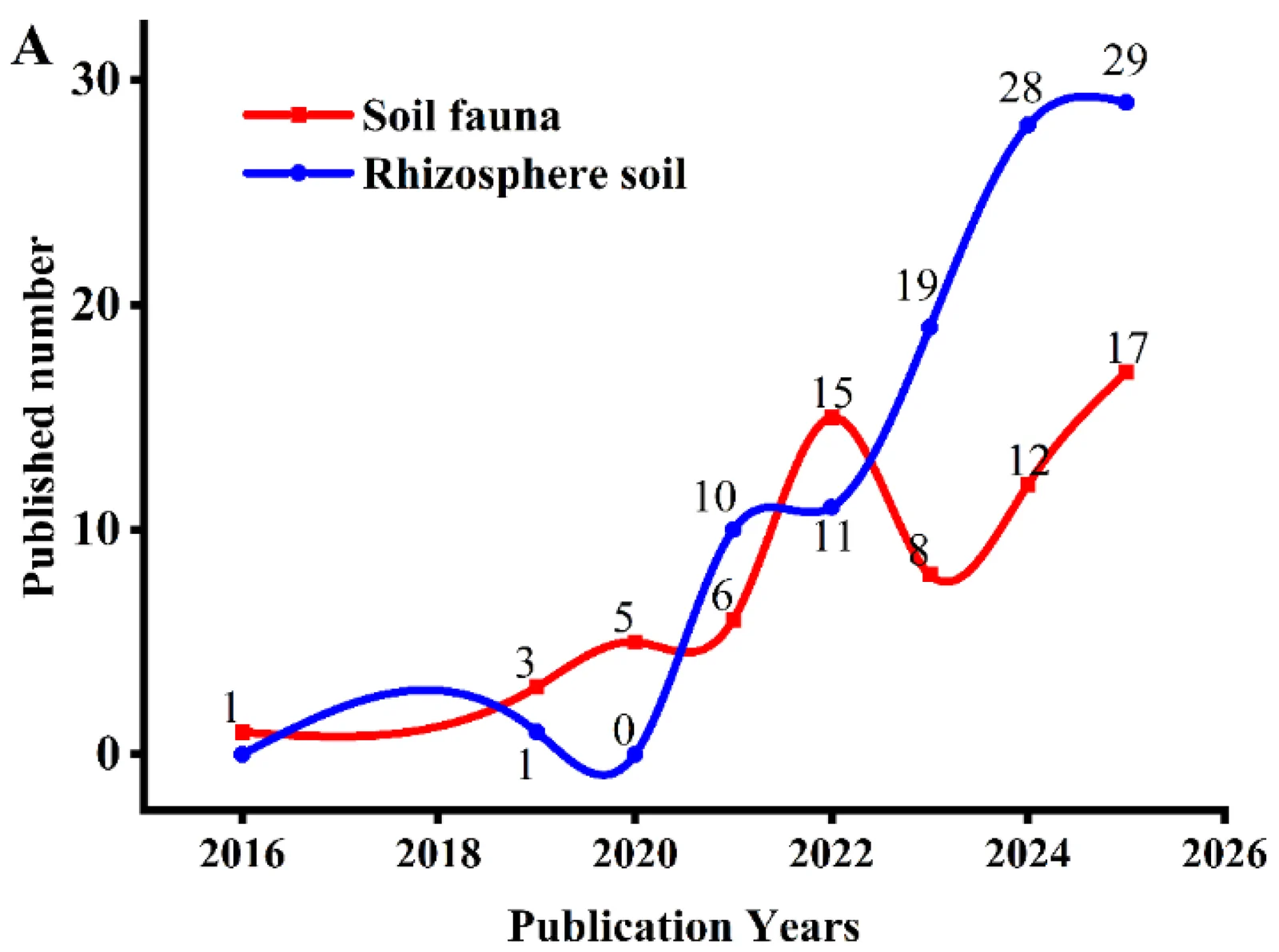

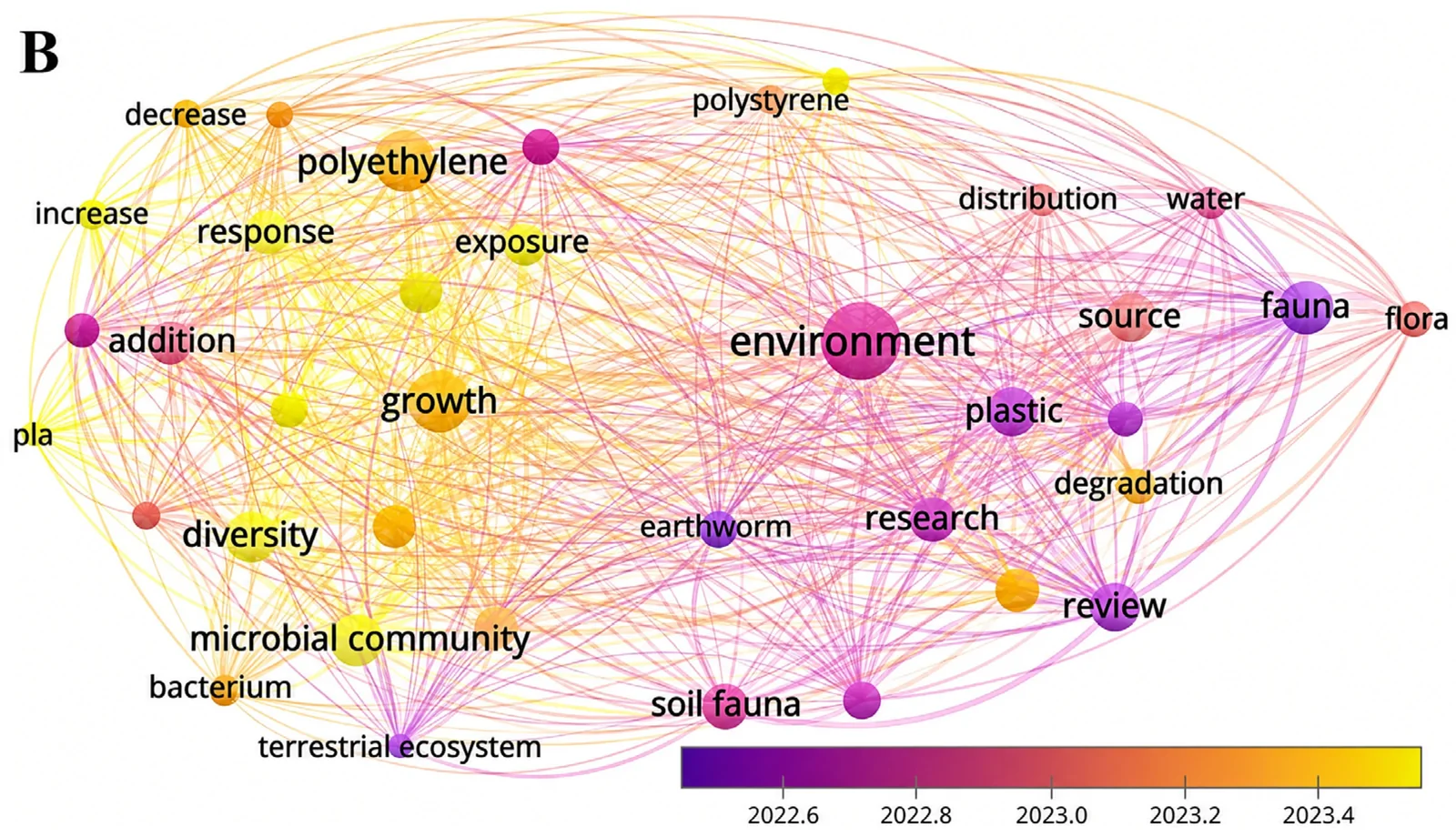
The published number of MPs and fauna or rhizosphere (**A**), and their study trends (**B**).

The Web of Science database was selected to retrieve articles on MPs in rhizosphere soil published between 1 January 2016 and 10 February 2026. Bibliometric network analysis was performed using VOSviewer (version 1.6.18.0), a freely available software tool for constructing and visualizing bibliometric networks. The analysis was conducted following a three-stage procedure: data retrieval, network construction, and visualization and exploration. In the first stage, relevant publications were retrieved from the Web of Science Core Collection database using a predefined search strategy. Second, the exported data were imported into VOSviewer(version 1.6.18.0) to construct the network. Finally, the generated networks were visualized and explored. VOSviewer provides three main visualization types: network visualization, overlay visualization, and density visualization. The resulting maps were then analyzed to identify major research themes, intellectual structures, and emerging trends. The inclusion criteria for this review were as follows: studies were required to (1) be relevant to environmental science, (2) primarily investigate the effects of MPs on rhizosphere soil, (3) be published in peer-reviewed journals, (4) provide a comprehensive description of the research methodology, and (5) be written in English. Figure 1A shows that 63 (search terms: “microplastic” and “soil fauna”) and 98 (search terms: “microplastic” and “rhizosphere soil”) related papers have been published from 2016 to 2026 (database: Web of Science). Search terms (“microplastic” and “Soil fauna” or “Rhizosphere soil”) exhibit trends in which hot topics, including earthworm, fauna, resource, and distribution, change into diversity, growth, microbial community, and response (Figure 1B). Therefore, this review mainly focuses on (1) the effect of MPs on soil properties as well as introduces the source, migration, and main degradation pathways of MPs in soil; (2) the changes in enzyme activity, microbial community, and functional gene mediated by MPs in rhizosphere soil; and (3) the response mechanism of crop and its soil fauna under MP stress. Notably, this work is the first to comprehensively outline the role of viruses in mediating the effects of both nondegradable and biodegradable MPs on soil carbon sequestration potential, which facilitates a better understanding of the interaction mechanisms between MPs and bacteriophages in soil. Finally, this review summarizes that MPs inhibit plant growth by inducing oxidative stress, interfering with nutrient uptake, disrupting photosynthesis and carbon metabolism, and altering plant hormone networks, which is of great value for assessing the ecological risks of MPs to plants.

## 2. Source, Migration and Main Degradation Way of MPs in Soil

MPs in farmland soils originate from various sources, among which residues of conventional plastic mulch films (predominantly PE) are considered a primary contributor. China, as a major agricultural country, consumes substantial amounts of agricultural film. Research on MPs in Russia has grown considerably in recent years, primarily focusing on freshwater ecosystems. Filimonova et al. [13] analyzed MPs in the Podolsk-Myachkovsky aquifer near Zvenigorod (Moscow region), identifying various polymers—including polyethylene, polyurethane, and polycarbonate—via Raman spectroscopy. Malygina et al. [14] conducted the first systematic survey of MPs in surface waters of six lakes in southern Siberia (including the Altai Mountains and the West Siberian Plain), reporting concentrations ranging from 4 to 26 MPs/L, with fragments and films as the dominant morphologies. Solodkova et al. [15] focused on MPs in bottom sediments of Lake Baikal for the first time, finding that fibers (predominantly polyester) were the main type, while fragments were composed mainly of alkyd resin (66%), polyvinyl alcohol (32%), and polyvinyl chloride (2%). Karnaukhov et al. [16] employed a multi-media approach and detected microplastics in surface water, the water column, sediments, macrophytes, macroinvertebrates, fish, and winter ice and snow in a bay of Lake Baikal. The polymer types included common polymers such as polypropylene and polystyrene, as well as rare polymers like alkyd resin and polyvinyl alcohol. European research on MPs holds a leading position globally, covering the entire land–sea continuum from rivers to deep-sea sediments. Additionally, the European Union has made the most systematic progress in policy regulation and health risk assessment. A study quantified MPs in soft sediments of European coastal waters, revealing that seawater temperature and salinity significantly influence polymer composition and deposition patterns, and further uncovering the interactions between MPs and macrobenthic communities [17]. Research in the United States has shown an accelerated transition from environmental monitoring toward health effects and policy legislation, with multiple federal legislative proposals and health-related studies attracting widespread attention in 2025. Using a Lagrangian particle tracking model coupled with a high-resolution circulation model, a study found that riverine MPs are a major contributor to pollution in the northern Gulf of Mexico, forming a significant accumulation zone west of the Mississippi River delta that overlaps with critical marine habitats, such as those of Kemp’s ridley sea turtles, red snapper, and bottlenose dolphins [18]. Teixeira et al. [19] found that biofouling can alter MP buoyancy and reshape shelf transport; small biofouled particles (0.001 mm) have a shelf residence time (31–34 days) approximately twice that of non-biofouled particles (17–19 days). During environmental transport, MPs readily form composite contaminants with other soil pollutants through van der Waals forces, electrostatic interactions, and surface complexation [20]. Such combined pollution behavior increases both the environmental risks and the complexity of MP transport. Biological disturbance is another important factor promoting MP migration in soil. The activities of soil fauna, such as earthworms, can transport MPs within the soil profile, thereby accelerating their vertical migration.

The abiotic degradation of plastics in the environment primarily involves photodegradation, thermal oxidation, and mechanical forces, with photodegradation being the most prevalent abiotic pathway [21]. Chemical degradation of MPs occurs through oxidation reactions facilitated by light, heat, or other chemical agents. This process breaks down the molecular chains, resulting in the formation of degradation products. The main types of chemical degradation are photooxidation and thermal oxidation [22]. The general mechanism of oxidative degradation of MPs can be divided into four stages: (1) chain initiation. Under light or heat exposure, polymers undergo dehydrogenation, resulting in the formation of macromolecular free radicals. (2) Chain propagation. In this stage, chain reactions occur in which macromolecular free radicals combine with oxygen to form polymer peroxy radicals. These then abstract hydrogen from another polymer molecule, producing hydroperoxides and new macromolecular radicals. (3) Chain branching and proliferation. The hydroperoxides formed decompose into new free radicals, which then abstract hydrogen from polymer molecules to create additional macromolecular compounds, thereby accelerating the oxidation process. (4) Chain termination. Finally, the free radicals either combine with each other or fail to undergo further reactions, leading to termination of the process and the formation of inactive products [23,24].

Value  added reaction (1–2):

(1) PH + X·→ P·+ XH

(2) P·+ O_2_ → PO_2_·

Chain branching reaction (3–7):

(3) PO_2_·+ PH → POOH + P·

(4) POOH → PO + OH

(5) PO·+ PH → POH + P·

(6) HO· + PH → H_2_O + P·

(7) PO·→ Multiple chain-breaking reactions

Chain termination reaction (8–9):

(8) P· + P· → P—P or P—H + P(—H)

(9) PO_2_· + PO_2_· → Inactive product

(Note: PH, P·, X·, XH, O_2_, PO_2_·, POOH, OH·, POH, PH, P—P and P—H represent polymer, macromolecule, and unspecified functional group, quenched product, oxygen, peroxy radical, polymer hydroperoxide, hydroxyl radical, alcohol, polymer chain, coupling termination, and disproportionation, respectively).

The biodegradation of plastics involves both physical comminution and subsequent microbial catabolism. Larger organisms can initiate fragmentation through ingestion, mastication, or maceration of plastic items, generating smaller particles that are more readily colonized and degraded by microbial communities. Once sufficiently fragmented, MPs typically undergo a four-stage microbial degradation sequence [25]: (1) biofilm formation. Microorganisms initially colonize the plastic surface by secreting extracellular polymeric substances, forming an adhesive matrix that anchors cells and provides the structural foundation for subsequent enzymatic action. (2) Physical erosion. Within the biofilm, microbial motility and localized enzymatic exo-actions compromise the structural integrity of the polymer matrix. As the polymer network loosens, fine particles detach, thereby increasing the specific surface area available for further degradation. (3) Enzymatic oxidation and chain scission. Enzymes such as laccases (oxidative) and esterases (hydrolytic) are released into the biofilm microenvironment. Through oxidation, dehydrogenation, and hydrolysis reactions, these enzymes cleave carbon–carbon and ester bonds within the polymer backbone. This enzymatic cleavage generates oligomeric and monomeric intermediates while simultaneously releasing any embedded plastic additives, representing the critical depolymerization phase [26]. (4) Uptake, assimilation, and mineralization. The resulting low-molecular-weight compounds and released additives are subsequently taken up by microbial cells and utilized as carbon and energy sources. Through intracellular metabolic pathways, these substrates are converted into microbial biomass and inorganic end products (e.g., CO_2_, CH_4_, and H_2_O), thus completing the mineralization cycle [27].

## 3. The Impact of MPs on Soil Properties

### 3.1. Effect of MPs on Soil Bulk Density and Pore Structure

The density of MPs is generally lower than that of soil mineral particles. Their substantial accumulation in soil can significantly reduce both the bulk density (by increasing total porosity) and the particle density (by diluting the heavy solid phase with light plastic components) [28]. Zhou [29] reported that at an application rate of 50 g kg^−1^, the bulk densities of soils amended with 1 mm and 25-μm PE particles were 88.57% and 88.20% of that of the control (CK), respectively. Such alterations in bulk density inevitably reconfigure the soil pore network, manifesting as a marked increase in total porosity and a shift in pore-size distribution (e.g., enhanced macro-porosity at the expense of meso-porosity). This physical restructuring directly modulates the solid–liquid–gas (three-phase) proportions within the soil matrix, thereby critically influencing soil aeration capacity and the biophysical microenvironment for root development. Research has demonstrated that the introduction of MPs increases the proportion of macropores in soil, yet may simultaneously reduce pore connectivity, leading to discontinuous pore structures that impede fluid transport [3]. The morphology and particle size of MPs significantly influence their effects on soil pore architecture. Fibrous MPs tend to entangle with one another, forming a network-like structure, whereas granular MPs exist more as discrete units within the soil matrix. The impact of MPs on soil porosity is closely related to their particle size and concentration [30]. In soil physics, pores are not merely empty spaces; their nature and origin—whether textural (capillary) pores derived from particle packing, structural macropores formed by aggregation, or cracks induced by shrink–swell dynamics—determine contrasting functions in water retention, gas diffusion, and root penetration. The introduction of exogenous particles such as MPs may distinctly perturb these different pore domains, a nuance that bulk porosity measurements often fail to capture.

### 3.2. Effect of MPs on Soil pH

The impact of MPs on soil pH has been confirmed by multiple studies; however, the direction and magnitude of this effect vary depending on MP type and soil properties [3]. Boots et al. [31] showed that adding HDPE to a sandy clay soil with an initial pH of 6.9 reduced soil pH by 0.62 units. This decrease may be attributed to HDPE-induced alterations in soil cation exchange capacity and to enhanced proton release at exchange sites in the soil solution. In contrast, PLA was found to increase soil pH in sandy ginger black soil, with pH rising progressively with increasing PLA application rates, potentially owing to a reduction in soil organic matter content. A study examining polyethylene MPs of different particle sizes revealed that all three sizes significantly reduced soil pH, with nanoscale PE exerting the most pronounced effect—a phenomenon possibly attributable to the release of additives such as plasticizers and stabilizers incorporated during MP manufacturing [32,33]. Conversely, another study investigating PLA effects on wheat rhizosphere soil also reported a pH reduction [34]. The influence of MPs on soil pH is contingent upon both MP type and soil physicochemical characteristics. For instance, the addition of 10% PLA reduced the pH of subalpine meadow soil by 12.08%. Given that PLA is a polymer derived primarily from lactic acid, the release of acidic degradation intermediates may be the primary driver of this pH decline [35]. However, other studies have found that PLA exerted no significant effect on the pH of horticultural soils in Northern Ireland [31]. Similarly, Wang et al. [36] found that PBAT/PLA blends increased soil pH in purple soils of southwestern China. These seemingly contradictory findings suggest that the influence of MPs on soil pH likely results from a complex interplay of multiple factors, including MP type, application rate, and baseline soil properties.

### 3.3. Effect of MPs on Stability of Soil Aggregates

Soil aggregates are the fundamental units of soil structure, and their stability directly influences soil resistance to erosion and nutrient retention capacity [37]. MPs can influence aggregate stability through two primary pathways. On the one hand, as exogenous particles, MPs may disrupt the binding agents that stabilize existing aggregates. On the other hand, their surfaces can interact with soil organic matter via adsorption and other processes, facilitating cementation and thereby promoting the formation of new aggregates or enhancing the stability of pre-existing ones [38]. Research on PE of varying particle sizes indicates that all three size classes—millimeter, micrometer, and nanometer—significantly improve soil aggregate stability, with micrometer-sized PE exhibiting the most pronounced enhancement [37]. This phenomenon may be attributed to the ability of micrometer-sized particles to effectively bridge soil primary particles while maintaining sufficient specific surface area to interact with soil cementing agents. While enhanced aggregate stability might imply a protective effect against erosion, it is crucial to recognize that the net impact of MPs on soil erosion is not unidirectional but rather reflects a dynamic trade-off between structural protection and hydrological disruption. The physical entanglement and surface coverage by MPs can indeed increase soil resistance to raindrop impact and overland flow [38]. However, these beneficial effects may be counterbalanced—or even negated—by concomitant adverse hydrological modifications. Specifically, the accumulation of MPs, particularly small-sized particles, can physically clog conducting pores and, owing to their inherent hydrophobicity, significantly intensify soil water repellency. These combined effects drastically reduce infiltration capacity, thereby promoting rapid surface runoff generation. Under such conditions, elevated runoff exerts greater hydraulic shear stress, substantially heightening the susceptibility of the soil surface to planar erosion [37]. Consequently, we propose that the ultimate effect of MPs on erosion critically depends on MP particle size, concentration, and the resultant balance between aggregate protection and runoff-driven erosive forces.

### 3.4. Effect of MPs on Moisture Transport in Soil

The influence of MPs on soil water transport is multifaceted and complex. First, the inherent hydrophobicity of MPs may intensify soil water repellency, thereby reducing infiltration rates and diminishing water availability [39]. Additionally, macropores created by MP incorporation can temporarily increase the saturated hydraulic conductivity of soil [40]. Nevertheless, over time, the migration and redistribution of MPs may induce pore clogging, consequently decreasing hydraulic conductivity. Notably, the effect of MPs on soil water transport is closely governed by the degree of surface aging [41]. Upon aging, the abundance of oxygen-containing functional groups on MP surfaces increases, while their hydrophobicity diminishes, thereby modifying their influence on soil hydraulic properties. Furthermore, both the concentration and distribution uniformity of MPs can influence the spatial heterogeneity of soil moisture, with elevated concentrations potentially inducing even greater spatial variability [40].

### 3.5. Effect of MPs on Soil Nutrients Cycling

The impact of MPs on soil carbon cycling is primarily reflected in their effects on soil organic carbon mineralization, dissolved organic carbon (DOC) content, and organic carbon stability [42]. The addition of MPs may influence the mineralization process of soil organic carbon by altering microbial activity and community structure [43]. Studies have shown that the addition of LDPE and PP can effectively suppress cumulative soil CO_2_ emissions [44], suggesting that MPs may reduce soil microbial activity or alter their metabolic pathways, thereby decreasing the decomposition of soil organic carbon. However, the effect of MPs on organic carbon mineralization varies depending on their biodegradability. PHAs strongly stimulate microbial activity and accelerate the decomposition of soil organic carbon, increasing decomposition rates by 200% to 250% and releasing substantial amounts of carbon dioxide into the atmosphere [45]. In contrast, PLAs degrade slowly in soil under ambient temperatures and exert a weak inhibitory effect on the decomposition of existing soil organic carbon, reducing decomposition rates by approximately 5% to 22% [45]. The effect of MPs on soil DOC also exhibits particle size specificity. Most studies have found that biodegradable MPs increase soil DOC content while decreasing soil dissolved organic nitrogen (DON) content [46]. Zhou et al. [47] reported that the addition of 10% PHBV increased soil DOC content by 54-fold and reduced DON content by 66%. Research has shown that millimeter- and micrometer-scale PEs significantly increase soil DOC content, whereas nanoscale PE significantly reduces it [46]. This discrepancy may be attributed to the differential effects of MPs with varying particle sizes on microbial communities. Millimeter- and micrometer-scale MPs significantly reduce the relative abundance of microorganisms associated with labile carbon utilization (e.g., Proteobacteria), while nanoscale MPs significantly increase the relative abundance of microorganisms involved in more stable carbon utilization (e.g., Penicillium), thereby affecting the balance between DOC production and consumption [33].

The effects of MPs on soil nitrogen and phosphorus transformations are complex and dependent on concentration and polymer type. Research on PE of different particle sizes indicates that all three size classes of PE significantly reduce soil ammonium nitrogen content, with millimeter- and micrometer-scale PE exhibiting more pronounced reductions. Both millimeter- and micrometer-scale PEs significantly decrease soil nitrate nitrogen content, whereas nanoscale PE shows no significant effect on nitrate nitrogen content. In contrast, PLA significantly increases the content of ammonium nitrogen, nitrate nitrogen, and total phosphorus in rhizosphere soil [48]. These contrasting results suggest that the effects of MPs on soil nutrient transformation may depend on MP type, particularly its biodegradability. PLA may release nutrients or stimulate microbial activity during decomposition, thereby promoting nutrient transformation, whereas PE may reduce nutrient availability through nutrient adsorption or alterations to microbial communities.

## 4. The Response Mechanism of Soil Microorganisms and Viruses Mediated by MPs

MPs substantially modify soil microbial community structure, manifesting as reduced bacterial and fungal diversity and enhanced dissemination of antibiotic resistance genes [49]. The plastisphere—a distinct biofilm colonizing MP surfaces—creates a unique ecological niche that critically modulates the ecological functions of the associated microorganisms [50]. As pivotal regulators of soil microbial communities, viruses profoundly influence soil carbon cycling and nutrient transformation in MP-contaminated environments, primarily through host cell lysis and the carriage of auxiliary metabolic genes [51].

### 4.1. The Impact of MPs on Rhizosphere Bacterial Communities

#### 4.1.1. Effect of MPs on Bacterial Community Structure and Diversity

A growing body of evidence has demonstrated that MPs can modulate the rhizosphere bacterial communities of plants. Zhang et al. [34] reported that PLA significantly reduced the richness and diversity of microbial communities in wheat rhizosphere soil, with the most pronounced reductions occurring at high concentrations (1 g/kg) and with large particle sizes (4000 μm). Notably, the number of unique genera in the high-concentration MP treatment group was significantly greater than that in the low-concentration group, underscoring the substantial impact of MP concentration on bacterial community structure [52]. Such a decline in richness—particularly under high-dose and large-particle conditions—may compromise the functional redundancy of the rhizosphere microbiome, potentially weakening the soil’s buffering capacity against environmental stresses, a key metric of soil biological effectiveness. A study on HDPE demonstrated that the rhizosphere bacterial community of cotton under HDPE treatment comprised 54 phyla and 472 genera, representing fewer taxa compared to soil treated with sterile water. Analyses of α- and β-diversity, together with ANOSIM and Adonis tests, revealed that HDPE-MPs significantly reduced bacterial community richness and altered community composition [53]. Such compositional shifts carry pedogenic implications, as they suggest altered microbial resource-utilization strategies, which could affect soil aggregate formation and root-derived carbon turnover—both processes intimately linked to soil development. A study on the combined pollution of PE-MPs and cadmium found that MP addition reduced the relative abundance of nitrogen-fixing Proteobacteria by 19.93%, whereas the relative abundance of nitrogen-cycling Proteobacteria and Actinomycetes increased with rising Cd concentrations [54]. The decline in nitrogen-fixing taxa points to a potential reduction in biological nitrogen input, which may exacerbate nitrogen limitation in contaminated soils. Conversely, the enrichment of Actinomycetes—known for heavy metal tolerance and recalcitrant organic matter degradation—suggests an adaptive response that could influence long-term soil fertility and metal immobilization. MPs exert a marked selective pressure on the composition of rhizosphere bacterial communities. Fei et al. [55] found that the addition of 1% PE significantly increased the relative abundance of taxa such as Sphingomonadales, Xanthomonadales, Propionibacteriales, Chitinophagales, Sphingobacteriales, and Flavobacteriales, while significantly reducing the relative abundance of unclassified Actinobacteria, Betaproteobacteriales, Myxococcales, and Gemmatimonadales. Many of the enriched orders are known for their capacity to degrade aromatic hydrocarbons and polymers, suggesting that PE addition may select for taxa with specific catabolic capabilities—a phenomenon that could accelerate the weathering of organic matter and alter humus composition, thereby affecting core pedogenic functions. Huang et al. [56] also observed that the addition of low-dose (0.076 g/kg) PE film (2 mm × 2 mm) to soil significantly reduced the abundance of Actinomycetes, possibly due to the tendency of certain biodegradable Actinomycetes to accumulate on MP surfaces. This reduction—contrasting with the enrichment reported in other studies—underscores the dose- and substrate-dependent nature of MP effects. A decrease in Actinomycetes could diminish their contributions to disease suppression and chitin degradation, thereby affecting the soil’s biocontrol potential. In contrast, Ren et al. [57] found that in soil amended with PE, the dominant microbial community shifted from Proteobacteria to Actinobacteria. Rather than a mere taxonomic swap, this shift carries pedogenic significance: Actinobacteria are proficient in decomposing recalcitrant polymers and participating in humification, which may be favored under PE-amended conditions where labile carbon is limited. Concurrently, the retreat of Proteobacteria—many of which are copiotrophic and involved in denitrification or plant-growth promotion—could alter nitrogen dynamics and root-signaling pathways, with cascading effects on soil biological effectiveness.

Additionally, PE treatment reduced the abundances of Acidobacteria, Nitrospirae, and Bacteroidetes, while the abundances of Proteobacteria—along with Acidobacteria and Bacteroidetes—continued to decline with prolonged exposure [58]. The progressive loss of Acidobacteria and Nitrospirae—the latter being key nitrifiers—suggests a gradual deterioration of nitrification capacity and carbon-cycling versatility, which over time may compromise soil fertility and aggregate stability, both critical indicators of soil genesis and health. The nitrogen-deficient conditions resulting from microbial nitrogen fixation during PHBV degradation may stimulate the proliferation of Chloroflexi, as this phylum typically dominates in nutrient-poor environments with low nitrogen availability [59]. This proliferation, while ecologically plausible, also implies that PHBV-driven nitrogen immobilization could shift the system toward a more oligotrophic state, thereby altering the balance between mineralization and immobilization—a fundamental pedochemical process. Proteobacteria may also participate in PHBV degradation, potentially enabling them to become a dominant bacterial group. Their enrichment in this context, however, may serve a dual role: contributing to polymer breakdown while potentially competing with plants for mineral nitrogen, thus modulating the net rhizosphere effect on crop nutrition. Different MP types elicit responses from distinct microbial communities. Sun et al. [60] observed that PLA addition significantly altered the microbial community structure of paddy soil, particularly affecting the genera Bradyrhizobium and Desulfobaca. Both MP concentration (0.3% and 1%) and exposure duration (10 d and 20 d) were identified as important factors influencing community structure. In paddy soils, shifts in Bradyrhizobium (a symbiotic N-fixer) and Desulfobaca (a sulfate-reducer) directly impact nitrogen fixation and anaerobic carbon mineralization, respectively; such changes could reshape the redox-driven pedogenic processes in flooded systems, affecting methane emissions and nutrient availability. Conversely, Chen et al. [61] found that the addition of 2% PLA had no significant effect on soil microbial community structure, a result that may be attributed to the adaptive buffering capacity of soil microbial communities. This insensitivity, however, does not negate ecological relevance; rather, it underscores that the magnitude of MP-induced community shifts depends on soil type, background microbiota, and exposure history—factors that are themselves products of pedogenesis and should be considered when evaluating the biological effectiveness of MP pollution.

Collectively, the findings discussed above indicate that MP-driven changes at the phylum or genus level are not merely statistical artifacts but may signify fundamental disruptions to soil nutrient cycling, organic matter transformation, and microbial interaction networks. We contend that interpreting these shifts through a pedogenic lens—that is, linking them to processes such as humification, nitrification, aggregate formation, and pathogen suppression—offers a more meaningful evaluation of the ecological and biological repercussions of MP contamination. This approach effectively moves beyond descriptive taxonomic inventories toward a functionally oriented pedo-microbiological framework.

In natural environments, the assembly of bacterial communities is governed by both stochastic processes (e.g., ecological drift and dispersal limitation) and deterministic processes (e.g., environmental selection and interspecific interactions) [62]. The introduction of MPs alters the balance between these two assembly mechanisms, consequently modulating community composition. Studies have demonstrated that bacterial community assembly within the plastisphere is predominantly governed by stochastic processes; however, the contribution of deterministic processes is significantly greater than that in the surrounding bulk soil [63]. This suggests that MP surfaces create an environmental filtering effect—offering distinct physicochemical conditions compared to the bulk soil—whereby only bacteria possessing suitable adaptive traits can successfully colonize [64]. Pronounced differences exist between the bacterial co-occurrence networks on MP surfaces and those in soil: the plastisphere network typically exhibits lower complexity and stability, yet higher modularity [63]. This structural divergence indicates that bacterial interspecific interactions within the MP microenvironment have been altered, potentially shifting from mutualistic associations toward intensified competition. Recent research reveals that MPs can render the interaction network of soil microbial communities more complex, even increasing network connectivity by a factor of 2–5 [65]. Furthermore, high concentrations of MPs (e.g., 7% *w*/*w*) can induce sharp declines in fungal and protist diversity and may simultaneously promote the proliferation of pathogenic microorganisms, thereby exacerbating the risk of crop diseases. Consequently, a critical question emerges: does the observed increase in network complexity confer greater resilience against external perturbations, or does it instead elevate the risk of systemic collapse due to the heightened vulnerability of keystone species? Resolving this question represents a pivotal direction for future research.

#### 4.1.2. Effect of MPs on Soil Enzyme Activity and Microbial Functional Genes

MPs can directly interfere with soil enzyme function through their physicochemical properties. Owing to their large specific surface area, MPs may adsorb soil enzyme molecules via electrostatic and hydrophobic interactions [66]. Such adsorption can alter the spatial conformation of enzyme proteins, masking or disrupting their active centers and thereby reducing the efficiency of enzyme-catalyzed reactions. Additionally, chemicals released from MPs may directly act on enzyme molecules, inhibiting their activity. Some studies have shown that nanoscale MP particles, owing to their small size, may even penetrate microbial cells and interfere with intracellular metabolic activities, thereby affecting enzyme synthesis and secretion [67]. The effects of different MP types on soil enzyme activity vary, with both the magnitude and direction of influence depending on their intrinsic characteristics. Multiple studies have found that biodegradable MPs (e.g., PLA) typically exert more pronounced effects on soil enzyme activity than non-biodegradable counterparts (e.g., PE, PP) [68]. This is attributable to the greater susceptibility of biodegradable MPs to microbial utilization as carbon sources, causing greater disturbance to the soil microenvironment during degradation. Exposure to high concentrations (>10%) of MPs generally leads to stronger inhibition or stimulation of soil enzyme activity [69]. Particle size is a key factor influencing the activity of soil carbon- and nitrogen-acquiring enzymes: smaller particles possess larger specific surface areas, enhancing their interactions with microorganisms and enzyme molecules [70]. Moreover, MPs of different shapes, such as fibers and fragments, may interfere with soil physical structure in distinct ways, resulting in divergent effects. Soil enzymes are primarily secreted by microorganisms and plant roots; therefore, microbial community structure directly determines the types and quantities of enzymes present [71].

**Figure 2:**
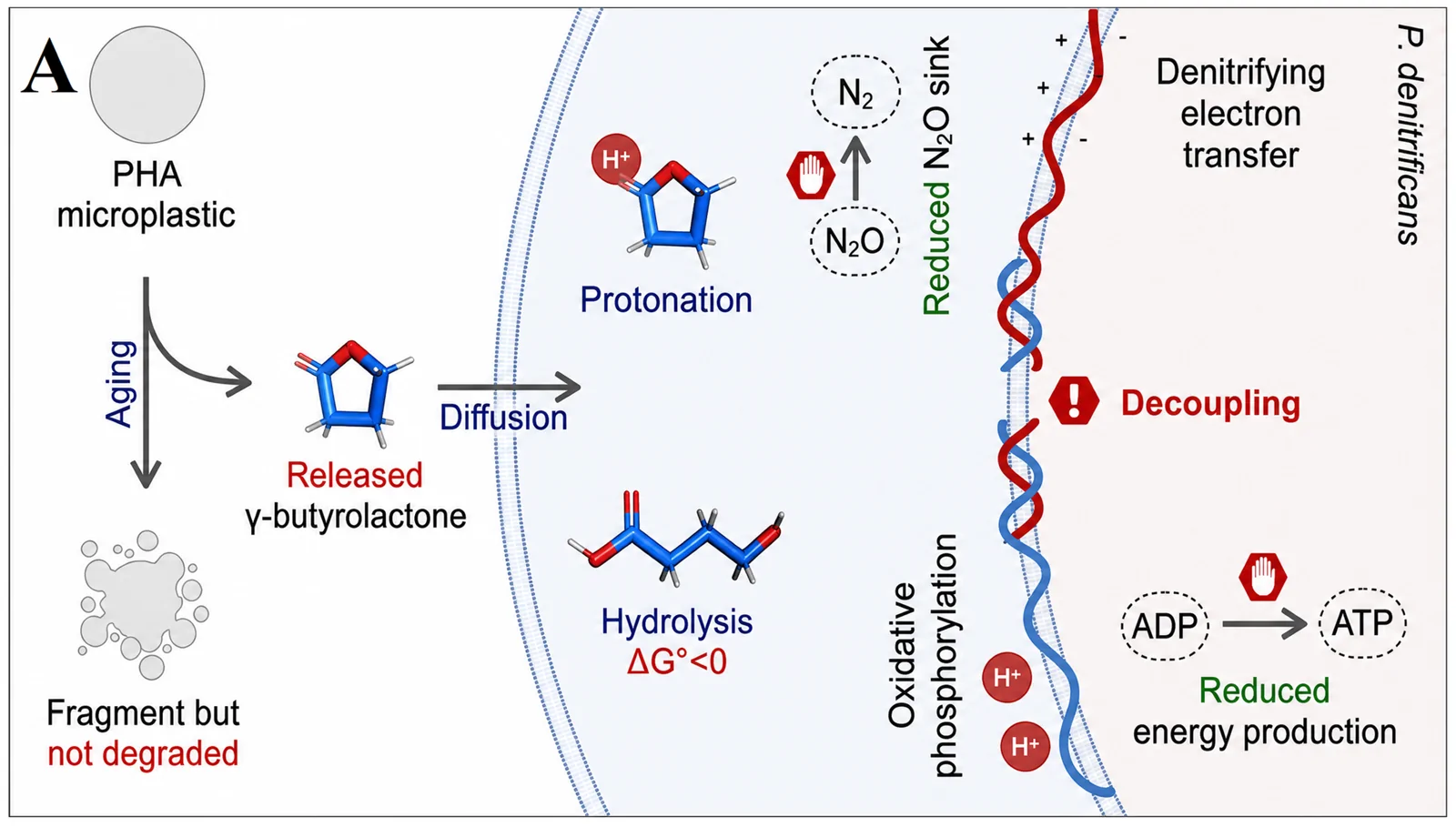

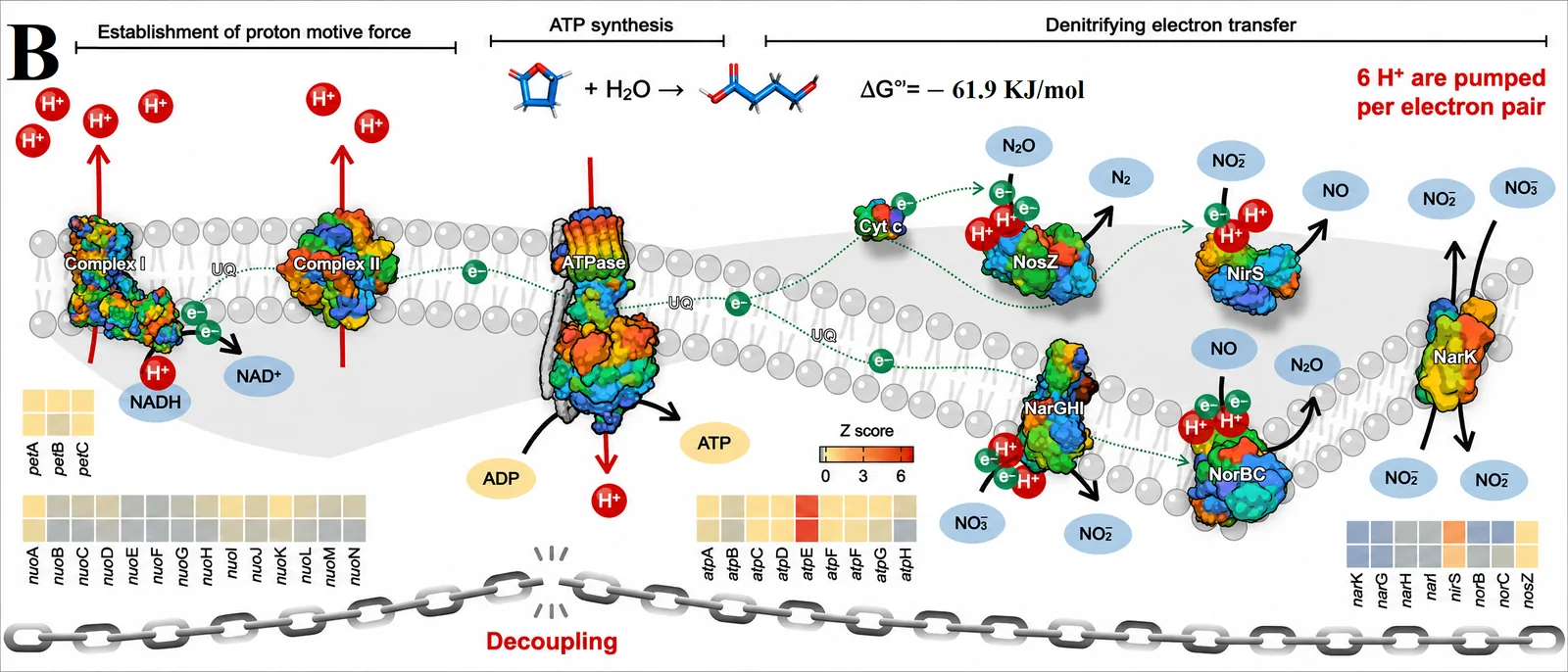
Hypothesized mechanism underlying the uncoupling of denitrifying electron transport from oxidative phosphorylation, induced by γ-butyrolactone during the aging of PHA. The periplasm is located above the phospholipid bilayer, whereas the cytoplasm resides beneath it. The schematic depicts the electrons and protons that are consumed by various enzyme complexes. Transcriptional expression levels of pivotal genes are presented in the heatmap, with the CK treatment shown in panel (**A**) and the γ-butyrolactone treatment in panel (**B**). Ubiquinone is abbreviated as UQ (Modified from Zhang et al. [79]).

The surfaces of MPs form unique “plastisphere” habitats that selectively enrich specialized microbial groups capable of adapting to their surfaces [72]. Such shifts in community structure inevitably led to changes in their functional outputs, namely enzyme secretion patterns [73]. Microbial functions are determined by the genes they harbor. Research has confirmed that MP addition can alter the abundance of functional genes associated with carbon and nitrogen cycling in soil [74]. For example, in the mountain ecosystem of Changbai Mountain, PE promoted the enrichment of carbohydrate-active enzyme functional genes in soils at mid- and low elevations [75]. Changes in functional gene abundance represent the direct source of altered enzyme activities. PHA has been shown to increase the activity of carbon-acquiring enzymes (β-1,4-glucosidase, β-glucosidase, and cellobiohydrolase), potentially because partial degradation of the MPs creates a carbon-rich environment in soil, stimulating microbial metabolism and enhancing enzyme activity [76]. Another study found that the biodegradable polymers PBAT and PLA increased the activities of catalase, dehydrogenase, and fluorescein diacetate hydrolase in soil, thereby enhancing soil microbial activity and influencing microbial carbon metabolism processes [,^77^]. Conversely, MPs may exert direct toxicity to aerobic microorganisms, consume oxygen during their degradation, or generate smaller plastic particles that reduce soil aeration, ultimately leading to decreased catalase activity [78]. Zhang et al. [79] investigated the effects of virgin and aged PHA (0.05–0.5% *w*/*w*) on the soil model strain *Paracoccus denitrificans*. Their findings indicate that γ-butyrolactone—which becomes protonated and spontaneously hydrolyzes in the periplasm—can disrupt the coupling between denitrifying electron transfer and oxidative phosphorylation by competing with ATPase for protons (Figure 2).

The introduction of MPs perturbs the normal metabolism of soil bacterial communities—a disruption that is directly reflected in the functional genes associated with carbon cycling. MPs can enhance the abundance or activity of genes involved in the utilization of labile carbon sources. As MPs, particularly biodegradable types, serve as alternative carbon substrates for microorganisms, genes related to the corresponding metabolic pathways may become enriched (Table 1) [80]. Functional prediction analysis indicates that MP treatment enriches genes involved in carbohydrate and amino acid metabolism [81]. Conversely, MPs may suppress genes responsible for the degradation of complex carbon compounds. When microorganisms exhibit a preference for utilizing relatively simple carbon sources such as MPs, their capacity to decompose native complex organic matter (e.g., cellulose) in soil may decline [82]. For instance, PVC has been found to significantly reduce the activity of β-glucosidase, an enzyme crucial for cellulose degradation. The impact of MPs on functional genes related to nitrogen cycling is particularly pronounced and complex, directly influencing soil fertility and greenhouse gas emissions [83]. MPs, especially the plastisphere formed by PE and PS, have been found to harbor abundant nitrogen-cycling functional genes, particularly those associated with denitrification. In strongly acidic soils, MPs may even serve as a refuge for denitrifying microorganisms, promoting their enrichment along with associated functional genes [84], and thus becoming a major source of greenhouse gas emissions. The plastisphere has been identified as a previously unrecognized source of N_2_O emissions in paddy soils. For example, LDPE can promote soil CH_4_ emissions by increasing the abundance of mcrA genes (involved in methanogenesis) [85] and can modulate soil N_2_O emissions by affecting the denitrification functional genes norB and narG [86]. PS may lead to significant increases in the activities of the nitrogen-cycling enzymes NAG and LAP [87]. The surfaces of MPs support the formation of high-density biofilms, providing an ideal microenvironment for horizontal gene transfer among bacteria. Research has found that PVC is significantly positively correlated with the relative abundance of various antibiotic resistance genes, including basS, rosB, and tetA [88]. MPs influence the dissemination of these antibiotic resistance genes by altering the presence and abundance of their bacterial hosts [88]. When MPs are present in combination with certain herbicides (e.g., imidacloprid), they can exert inhibitory effects on soil microbial populations and induce shifts in functional bacterial genera associated with nitrogen cycling, thereby altering the expression of functional genes. Such combined exposure may promote nitrogen fixation and denitrification while inhibiting nitrification [89].

### 4.2. The Effect of MPs on Rhizosphere Fungal Communities

#### 4.2.1. Effect of MPs on the Structure and Diversity of Rhizosphere Fungal Communities

The response of rhizosphere fungal communities to MP pollution is more pronounced than that of bacterial communities [52]. Research has shown that PEs increase the overall richness and diversity of soil bacterial communities while reducing those of soil fungal communities. Accinelli et al. [100] demonstrated that during a 12-month incubation period, the surface area of biodegradable MPs decreased across different soils, while the quantity of released fragments steadily increased. Among these fragments, 88.4% were associated with Aspergillus flavus, and 68% of the strains isolated from CFMPs were capable of producing aflatoxin. This indicates that burying biodegradable MPs in soil or applying compost containing CFMPs may compromise soil quality and elevate the risk of establishing toxigenic Aspergillus flavus populations in soil [100]. Studies have revealed that PLAs significantly affect the relative abundance of specific arbuscular mycorrhizal fungal (AMF) groups, such as Paraglomus and Glomus, indicating that PLA exerts selective effects on certain AMF species [101]. Another study found that PLA MPs at concentrations ranging from 0.1% to 10% significantly altered AMF community structure, with higher concentrations exerting greater effects. This contrasts with previous findings, as the relative abundances of other specific AMF taxa (Glomeraceae and Ambispora) were altered [102]. Research has demonstrated that fungi such as Penicillium and the pathogenic Alternaria are significantly enriched in the plastisphere. This selective enrichment directly alters fungal community composition. The richness and diversity of fungal communities in the plastisphere are generally lower than those in the surrounding soil [103]. For instance, Sun et al. [104] found that the Chao1 richness index of the plastisphere-associated fungal community was significantly lower than that of the surrounding soil. The fungal community in the plastisphere is not static but undergoes dynamic succession over time. In the early stages, opportunistic taxa capable of rapid growth preferentially colonize; over time, species poorly adapted to this environment are eliminated, leading to substantial shifts in community composition [105]. In bulk soil, fungal community assembly is often governed by deterministic processes such as environmental filtering by factors like pH and nutrients [106]. In the plastisphere, however, the role of stochastic processes is significantly enhanced, becoming the dominant force. This implies that which fungi survive and proliferate on MP surfaces is more influenced by chance factors and relatively less affected by traditional environmental factors such as soil nutrients [107].

Compared to fungal networks in soil, fungal co-occurrence networks in the plastisphere are typically smaller in scale and lower in complexity. This indicates that fungal interspecific interactions in the MP environment have been altered, with relationships among species becoming simpler and more unstable [104]. The presence of potential plant pathogenic fungi (e.g., Alternaria) in the plastisphere may increase the risk of crop diseases, posing a potential threat to agricultural ecosystems. For example, biodegradable MPs such as PLA and PBAT are more readily utilized by microorganisms during degradation, and their disturbance to fungal communities is often more significant than that of conventional PE and PS [105]. Fibrous MPs may directly affect fungal hyphal extension through physical entanglement and blockage. The ecological effects of MPs typically exhibit concentration- and time-dependency, with high concentrations and long-term exposure often leading to more pronounced impacts [105,108].

#### 4.2.2. Effect of MPs on Soil Enzyme Activity and Fungal Functional Genes

The impact of MPs on soil fungal enzyme activity represents a complex process through which MPs interfere with the normal physiological functions of fungi via direct and indirect pathways, thereby affecting the health of the entire soil ecosystem [109]. Upon encountering fungi, the physicochemical properties of MPs directly induce oxidative stress in fungal cells. In response to MP stress, fungi activate their endogenous antioxidant enzyme systems as a defense mechanism [110]. For instance, exposure to PS significantly increases the activities of superoxide dismutase and peroxidase in *Pleurotus tuber-regium* and *Lactarius deliciosus* during the early stages, serving to eliminate excess reactive oxygen species. However, when stress intensity exceeds the regulatory capacity of fungi, the activity of key enzymes such as catalase may become significantly inhibited under high stress conditions. Intense oxidative stress can trigger membrane lipid peroxidation, leading to elevated malondialdehyde content and increased tissue conductivity, indicating damage to cell membrane structure and loss of integrity [111]. MPs can interfere with the activity of key metabolic enzymes involved in fungal nutrient cycling, disrupting normal energy allocation and material metabolism. For lignin-degrading fungi, the presence of MPs can disrupt enzyme secretion patterns [107]. For example, polyethylene terephthalate fragments promote the secretion of laccase by Fusarium oxysporum but inhibit its production of manganese peroxidase and lignin peroxidase [112]. As lignin degradation represents the rate-limiting step in soil carbon cycling, such inhibition may slow the decomposition of organic matter and the release of carbon dioxide. MPs, particularly biodegradable types, serve as an exogenous organic carbon source and can alter the carbon-to-nitrogen ratio of soil [113]. This may disrupt fungal metabolism, prompting fungi to adjust their enzyme secretion profiles in response to this novel carbon source, thereby affecting inherent soil nutrient cycling pathways [10,33]. Srikanth et al. [114] described the enzymatic mechanisms employed by *P. ostreatus* to foster the hydrophilicity of plastics, thereby facilitating the breakdown of large polymer chains into smaller, more manageable molecules.

MPs also indirectly influence fungal enzyme activity by modifying soil physicochemical properties and the living environment of fungi. Changes in soil physical structure can create additional anaerobic zones, affecting the activity and enzyme secretion of aerobic fungi [46]. Chemical additives such as plasticizers and stabilizers incorporated during MP manufacturing are slowly released from the plastic matrix; these compounds may exert direct toxicity to enzyme proteins, inhibiting their activity [69]. Under MP stress, certain fungi are stimulated to develop degradation potential and respond by upregulating the expression of specific enzymes. Some fungi can degrade MPs through the secretion of specialized enzymes [115]. For instance, studies have shown that Fusarium vanettenii significantly upregulates the expression of genes encoding enzymes such as lipase and keratinase during the degradation of polyurethane and polyethylene terephthalate. These enzymes act synergistically to break down plastic polymers into smaller molecular compounds. Molecular docking simulations indicate that the lipases FvLIP1 and FvLIP2 can bind to plastic monomers through hydrophobic interactions and hydrogen bonding, providing a structural basis for their degradative capacity [112]. In environments, MPs often co-occur with other pollutants, resulting in combined effects [116]. When MPs are present in combination with herbicides such as imidacloprid, they exert inhibitory effects on soil microbial populations and induce shifts in functional bacterial genera associated with nitrogen cycling, thereby altering the expression of functional genes. Such combined exposure may promote nitrogen fixation and denitrification while inhibiting nitrification [70,89,115]. In summary, MPs affect soil fungal enzyme activity through multiple mechanisms, including the induction of oxidative stress, direct interference with metabolic enzyme activity, alteration of the soil microenvironment, and synergistic interactions with co-occurring pollutants. These effects collectively disrupt soil nutrient cycling and ecological functions.

### 4.3. Effect of MPs on Rhizosphere Soil Viral Communities

**Figure 3:**
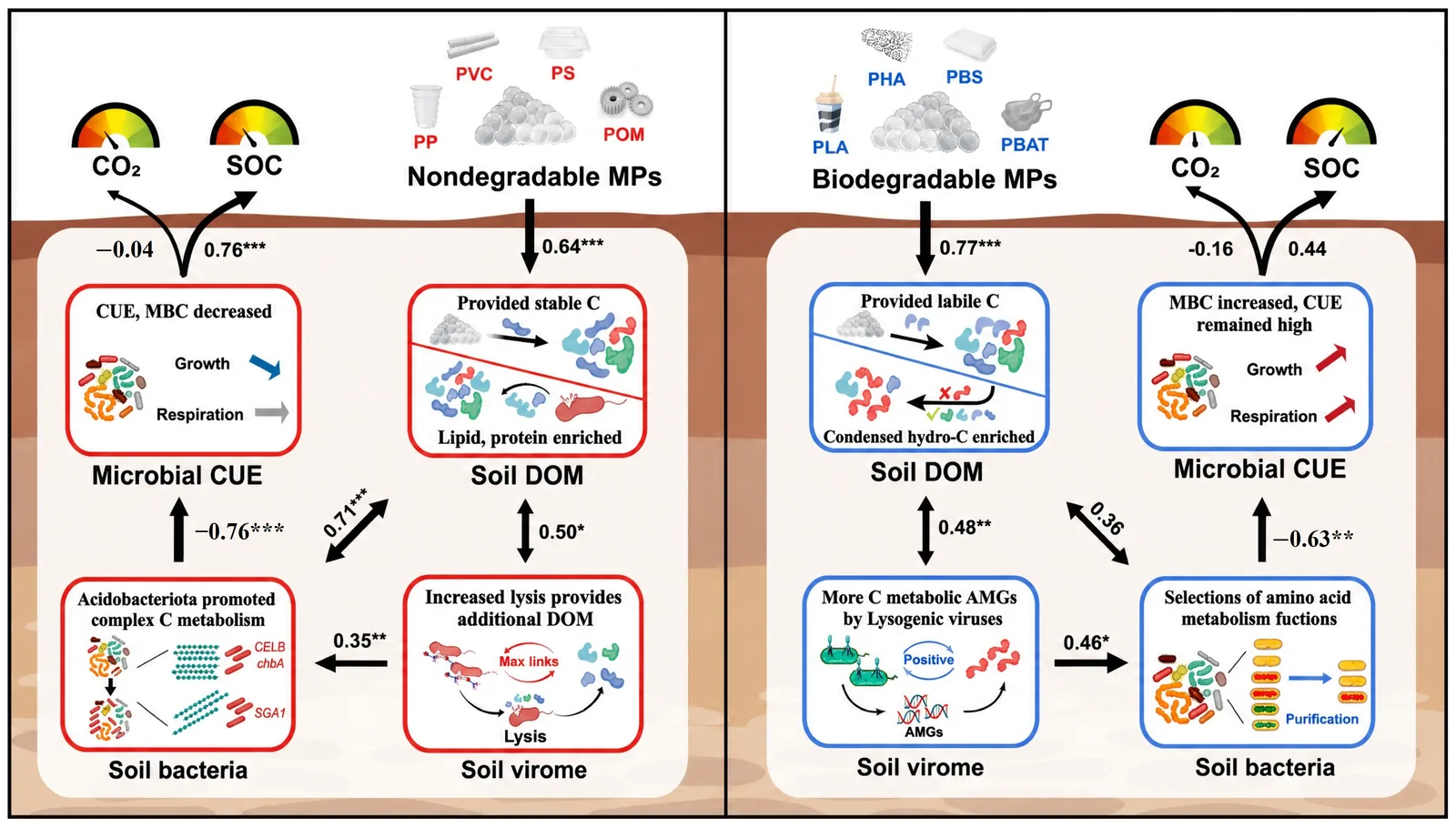
A schematic overview of how biodegradable versus nondegradable MPs affect the soil carbon cycle. Structural equation modeling was performed independently for each MP type in order to assess the relationships between MP addition, DOM, soil bacterial communities, virome, microbial CUE, SOC, and CO_2_ release. Standardized path coefficients are denoted by the numbers placed on the connecting lines, with significance levels marked by * (*p* < 0.05), ** (*p* < 0.01), and *** (*p* < 0.001). (Modified from Wang et al. [51]).

As the most abundant microbial entities in soil, viruses play an important regulatory role in MP-contaminated environments [51]. Research has shown that biodegradable MPs significantly reduce viral alpha diversity across all fertilization treatments, altering viral community structure, community resistance, and taxonomic turnover [117]. In contrast, conventional MPs have been found to alter the abundance of viral families primarily in manure-amended soils [118]. Studies on soil surrounding rural waste disposal sites have revealed that soil viral communities are predominantly composed of Siphoviridae (long-tailed bacteriophages) and Myoviridae (short-tailed bacteriophages). Biodegradable MPs are associated with increased alpha diversity of antibiotic resistance genes (ARGs), shifts in ARG community structure, and changes in community resistance, particularly at the transcriptional level. Notably, regardless of fertilization regime, biodegradable MPs significantly enriched high-risk ARGs and mobile genetic elements in soil [119]. Correlation analyses have highlighted the important role of lytic viruses in shaping high-risk ARGs, indicating that viruses represent an indispensable factor in the MP-promoted dissemination of resistance genes [120]. Viruses in soil play a central role in ecosystems by regulating the community structure, function, and metabolic activities of their host microorganisms [121]. Their mechanisms of action are complex, with profound implications for soil health, plant growth, and global biogeochemical cycles. MPs themselves are carbon-rich polymers that can serve as additional carbon sources for microorganisms. Viruses can regulate microbial community turnover and metabolism through lytic and lysogenic life cycles, thereby influencing the carbon fate of MPs. Under pollution from non-biodegradable MPs such as polyethylene and polyvinyl chloride, viruses tend to adopt a lytic strategy. Although this can alleviate carbon availability limitations by releasing lysates, it encourages microorganisms to allocate more carbon toward energy metabolism rather than growth, thereby reducing the carbon sequestration potential of soil microorganisms [51]. Under PLA pollution, viruses integrate more frequently into host genomes via lysogeny and utilize their auxiliary metabolic genes to enhance host carbon metabolism (e.g., in copiotrophic bacteria), thereby increasing the carbon sequestration potential of soil microorganisms (Figure 3) [51]. Compared to MPs themselves, their associated additives (plasticizers) may exert more pronounced effects on soil viral communities. Exposure to plasticizers such as diethyl phthalate can lead to a sharp increase in soil viral sequence abundance (a 3.15-fold increase), far exceeding that induced by PE and PVC. Such exposure significantly alters viral diversity, functional gene composition, and virus–host interactions and induces widespread prophage activation [121]. A key mechanism involves viral-encoded auxiliary metabolic genes, such as the gene encoding 3-oxohexanoyl lactonase, which has been found to be crucial for plasticizer degradation, directly facilitating microbial adaptation to and decomposition of these pollutants. The biofilm formed on MP surfaces provides a unique habitat for viruses and bacteria, greatly promoting the dissemination of ARGs [121]. MPs can serve as carriers for non-enveloped viruses, helping them maintain infectivity and undergo long-distance transport over extended periods. Research has identified Myoviridae and Siphoviridae as the predominant viral groups on MP surfaces, where their associations with hosts such as Proteobacteria and Firmicutes are closer than those observed on natural substrates such as stone and wood. Common antibiotic resistance genes and metal resistance genes have been detected in the genomes of viruses and their host bacteria on MP surfaces, providing direct evidence for phage-mediated horizontal transfer of these resistance genes [122].

## 5. Response of Soil Fauna Under MPs-Mediated

Although studies on the toxic effects of MPs on soil fauna remain relatively limited, earthworms have received comparatively more attention as representative soil organisms. However, the reported toxicity outcomes are not uniform; rather, they vary considerably depending on the physicochemical properties of the MPs and the surrounding environmental matrix. Polymer type plays a critical role: conventional non-biodegradable polymers (e.g., polyethylene, polypropylene) and biodegradable alternatives (e.g., PLA) differ in their additive formulations, surface hydrophobicity, and degradation intermediates, leading to distinct toxic profiles—the former primarily causing persistent physical damage and slow additive leaching, while the latter may release oligomers and other metabolites that interfere with earthworm homeostasis. Particle size determines the route and extent of biological interaction: nano-sized MPs, owing to their high specific surface area and tissue-penetrating capability, tend to provoke stronger oxidative stress and cellular toxicity, whereas micro-sized particles more often induce epidermal abrasion (especially at high concentrations ≥ 1.0 g/kg) and gut blockage, with markedly different dose–response thresholds [123]. Furthermore, aging significantly modifies the surface chemistry of MPs: UV irradiation or mechanical weathering increases oxygen-containing functional groups (e.g., carboxyl, hydroxyl), which not only enhances the vector capacity for co-existing heavy metals or organic pollutants but also accelerates the release of embedded additives, thereby amplifying indirect chemical toxicity [3]. Finally, soil conditions—including pH, organic matter content, and texture—influence the aggregation state, vertical mobility, and actual contact probability of MPs with earthworm skin and gut, as well as the burrowing behavior of the animals, thus modulating the effective exposure dose in situ. Against this background, the core injury mechanisms—epidermal damage compromising skin respiration and moisture retention [124], additive-induced chemical toxicity [3], overproduction of reactive oxygen species and consequent disruption of the cellular antioxidant balance [125], shifts in antioxidant enzyme activities [126], dysbiosis of gut microbiota that impairs digestion, absorption, and immune regulation [127], and trophic transfer along the food chain (e.g., earthworm-to-chicken) [128]—are all triggered at different intensities and thresholds depending on the interplay of polymer type, size, concentration, aging, and soil properties. Exposure concentration is not linearly correlated with effect intensity; at environmentally relevant levels (typically mg/kg), antioxidant enzyme systems (e.g., superoxide dismutase, catalase) may show adaptive upregulation, whereas only beyond a critical threshold does significant redox imbalance and oxidative damage to lipids, proteins, and DNA occur [126]. Consequently, the migration and biomagnification of MPs across the soil–plant–animal continuum are not universal but exhibit substantial variability among exposure scenarios [127]. Future risk assessments should therefore adopt a tiered, parameter-standardized framework to identify high-risk conditions and avoid overgeneralizing findings from a single experimental setup to the complex reality of field soils.

Owing to their vulnerability, soil fauna are widely used as model organisms for detecting the toxicity of pollutants such as MPs, polycyclic aromatic hydrocarbons, and pesticides. The factors influencing the toxic effects of MPs on soil fauna are complex and diverse, including particle size, abundance, shape, polymer type, additives, and degree of aging. The toxicity and underlying mechanisms of MPs on soil fauna primarily manifest in aspects such as feeding behavior, growth and development, oxidative stress, intestinal toxicity, and reproductive toxicity [127]. Earthworms are frequently employed due to their high soil ingestion rates and key role in nutrient cycling, but recent work also emphasizes collembolan and enchytraeid responses to MP exposure [126]. Toxic outcomes depend on a constellation of particle attributes—size, shape, polymer chemistry, surface aging, and associated additives—which together govern bioavailability, gut retention time, and interaction with co-contaminants such as polycyclic aromatic hydrocarbons or heavy metals [12,127]. For instance, fibrous MPs can entangle in the foregut, abrading tissues and obstructing peristalsis, whereas NPs more readily translocate into coelomic cavities and subcutaneous layers, eliciting systemic effects [127,128]. Mechanistically, MP ingestion disrupts feeding behavior, growth, reproduction, and survival through a combination of physical abrasion, oxidative stress, and molecular interference. In *Caenorhabditis elegans*, PS-NPs (~1 μm) trigger down-regulation of the DBL-1/TGF-β and DAF-16/insulin-like signaling pathways, manifesting as shortened lifespan and reduced body length [129,130]. Concurrently, MPs induce reactive oxygen species overproduction, initially up-regulating antioxidant defenses (SOD, CAT, GPx, GST) but eventually overwhelming enzymatic capacity and elevating MDA levels—a pattern documented in terrestrial snails at 0.71 g kg^−1^ MP exposure (−59.3% GPx; +58.0% MDA) [131,132]. Histopathological analyses further reveal intestinal mucosal erosion, villus rupture, and epithelial cell detachment in both annelids and nematodes, often accompanied by dysregulated expression of ion-transport genes (vha-6, opt-2, nhx-2) that disturb Ca^2+^ homeostasis and gut pH [127,133]. Beyond individual-level toxicity, MPs reshape soil–fauna–microbiome networks and ecosystem services. PVC fragments alter insect gut bacterial diversity, impairing lignocellulose digestion and nutrient uptake in phytophagous larvae [134], while earthworm-mediated bioturbation under PE and PP stress enhances tomato resistance to Helicoverpa armigera via induced chlorogenic acid and jasmonic acid production [135]. These findings underscore a dual role for soil fauna as both victims of MP pollution and potential bioremediators. Moving forward, integrating multi-omics approaches with field-realistic exposure scenarios will be essential to unravel the long-term ecological cascades of MP pollution and to develop strategies that harness soil fauna for sustainable soil health management.

## 6. Response of Plants Under Response of Plants Under MPs Stress

In soil, MPs can interact with plants through direct root uptake and indirect physical contact. MPs, particularly larger particles, tend to accumulate around plant roots and exert indirect effects by altering the microenvironment of the rhizosphere. Previous studies have demonstrated that submicron- and nanoscale plastics can enter plant cells. Nano-sized PE accumulates in the maize rhizosphere as well as in the roots, stems, and leaves of wheat [136,137]. PS particles of 80 nm and 1 μm are enriched in the root columns, stem vascular bundles, and leaf veins of rice tissues, with predominant accumulation in cell walls and intercellular spaces [138]. Submicron-sized PS particles can also enter the roots of wheat seedlings and be translocated to the vascular systems of stems and leaves [139]. Micro- and nanoplastics may further enter plant flowers and fruits; for instance, nano-sized PS particles have been observed to translocate from cucumber stems to leaves, flowers, and fruits. The uptake of micro- and nanoplastics by plants varies with particle size. Studies have shown that tobacco does not take up 100 nm PS particles but does absorb particles in the 20–40 nm range [140]. Additionally, fluorescent 0.2 μm PS particles can enter cells beyond the root cap mucilage [141], and 0.2 μm PS particles have been observed in the cell walls and vascular systems of root cortical tissues, indicating that nanoplastics enter the plant body via intercellular pathways, specifically the apoplastic transport system [142]. Consequently, MPs can influence plant growth and germination, alter the composition of root exudates, and modify the soil environment. MPs may also adhere to plant roots or directly enter plant tissues. Furthermore, they can enter the vesicles and hyphae of arbuscular mycorrhizal fungi, thereby affecting AMF colonization of plant roots.

**Figure 4:**
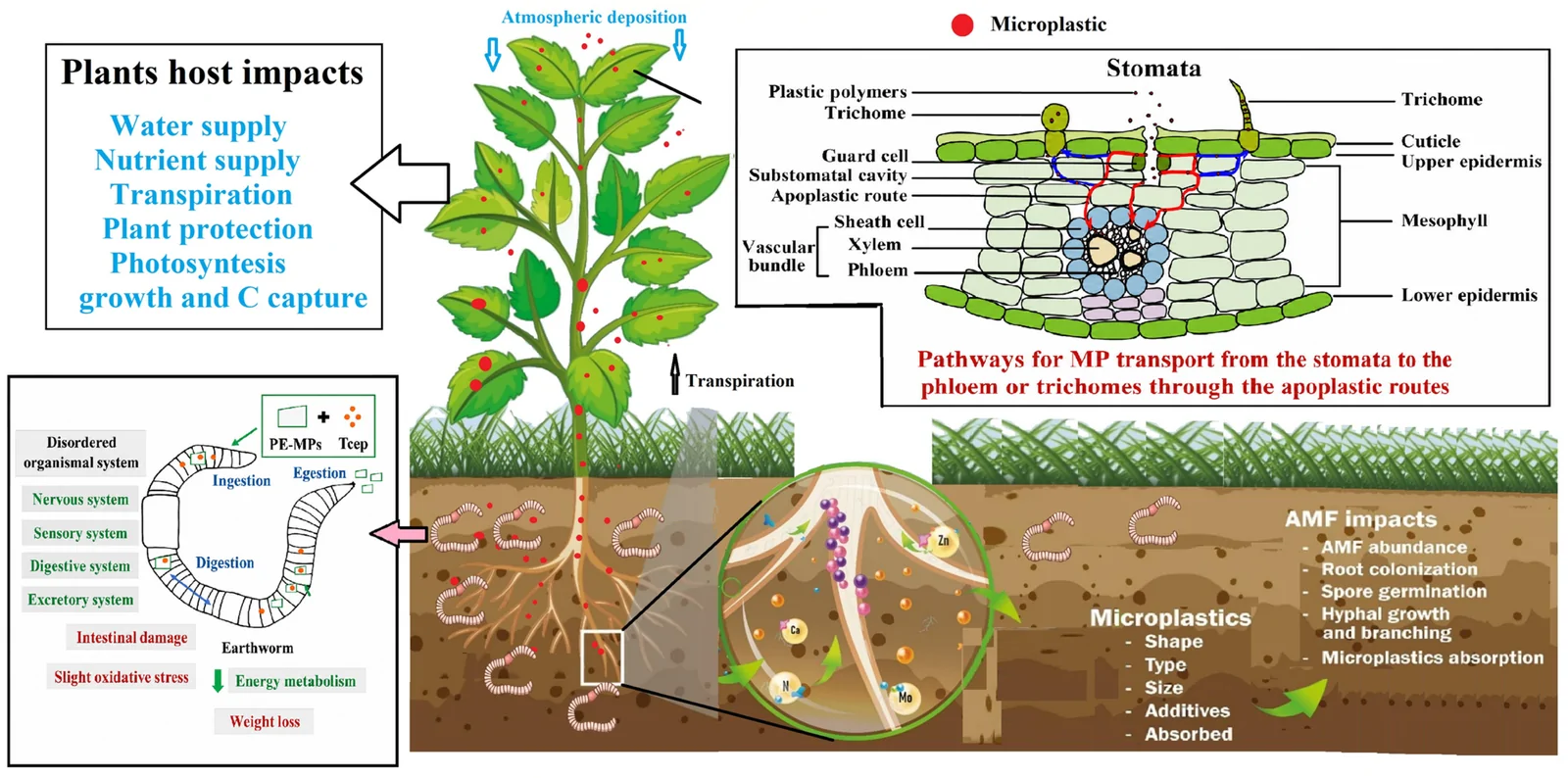
Response of soil–plant system under MP stress (Modified from Li et al. [12], Álvarez-Lopeztello et al. [134] and Cao et al. [135]).

The direct effects of MPs on plants include physical barriers and mechanical damage, induction of oxidative stress, interference with nutrient uptake, disruption of photosynthesis and carbon metabolism, and disruption of plant hormone networks [143,144,] (Figure 4). (1) Physical barriers and mechanical damage. MPs, particularly fibrous types, can form a dense network in soil that physically impedes the downward penetration and extension of plant roots. This can lead to abnormal root development, reduced root length, and decreased numbers of lateral roots, thereby limiting the efficiency and range of water and nutrient absorption [145]. As most MPs in the environment are formed through the long-term degradation of plastics, they typically exhibit small particle sizes and large specific surface areas [146]. They can adsorb onto seed surfaces, hindering the seed’s access to nutrients and water. Moreover, MPs can adhere to plant roots, further affecting plant metabolism [147]. Consequently, the presence of MPs in soil can delay seed germination, reduce germination rates and seedling survival, and significantly alter plant biomass, nutrient uptake, and root traits, thereby affecting AMF colonization. (2) Induction of oxidative stress. Oxidative stress represents the core biochemical mechanism through which MPs induce damage in plants. When plant roots perceive MP stress, they trigger a burst of reactive oxygen species within their tissues. Excessive ROS can attack biomolecules, leading to lipid peroxidation of cell membranes, protein inactivation, and DNA damage [148]. Although plants activate antioxidant enzyme systems (e.g., superoxide dismutase and catalase) as a defense response, excessive stress can still result in cellular damage or even cell death [149]. (3) Interference with nutrient uptake. MPs can severely disrupt the absorption of essential nutrients such as nitrogen, phosphorus, and potassium by plants [150]. Dan et al. [151] found that biodegradable PLAs with small particle sizes (25–38 μm) strongly inhibit plant nitrogen uptake. The underlying mechanism is that these small-sized MPs greatly stimulate soil microbial activity, causing microorganisms to rapidly immobilize ammonium nitrogen from the soil into their own biomass. Although nitrogen transformation proceeds rapidly, plants are unable to utilize the nitrogen sequestered by microorganisms, thereby entering a state of nitrogen deficiency. (4) Disruption of photosynthesis and carbon metabolism. MPs can be translocated to aboveground plant tissues, where they may reduce leaf chlorophyll content and damage chloroplasts—the key organelles for photosynthesis—leading to decreased photosynthetic rates [152]. Research on barley has shown that MPs significantly alter the activity of key enzymes involved in carbohydrate metabolism in both roots and leaves, disrupting the normal energy supply and carbon skeleton construction in plants [153]. (5) Disruption of plant hormone networks. Plant growth and development are precisely regulated by hormones; therefore, the intrusion of MPs can disturb the balance of endogenous hormones [154]. For example, in barley, MPs induce an abnormal increase in the content of cytokinins (such as trans-zeatin) in leaves, while significantly reducing the content of auxins (such as indole-3-acetic acid). Such hormonal imbalances directly affect normal plant growth and developmental processes [154]. Overall, MPs (<5 mm) and NPs (<100 nm or <1 μm) exhibit significant differences in plant absorption, transport, and toxicity due to their fundamental size disparity. NPs can readily penetrate key barriers such as plant cell walls and enter the interior, whereas MPs mainly remain on root surfaces or in intercellular spaces.

## 7. Conclusions and Perspective

This review has systematically examined the response mechanisms of crops and their associated soil fauna to MP stress, alongside MP-induced alterations in rhizosphere enzyme activities, microbial community structure, and functional gene expression. MP reduce bulk and particle densities, disrupt existing aggregates, and can form new organo-mineral associations. MPs alter bacterial metabolism and carbon-cycling functional genes, while fungal communities show even greater sensitivity, with key nutrient-cycling enzymes being inhibited. Through lytic and lysogenic cycles, viruses modulate microbial turnover and thus influence the carbon fate of MPs. MPs can affect plant growth via multiple pathways, including physical barrier damage, oxidative stress, impaired nutrient uptake, photosynthetic disruption, and hormonal imbalance; they also affect soil fauna by disrupting feeding, growth, reproduction, gut health, and inducing oxidative stress.

Despite these insights, significant knowledge gaps persist. A critical issue is the environmental relevance of applied MP concentrations. Most laboratory studies use doses at percentage levels (e.g., 0.5–10% *w*/*w*), which are orders of magnitude higher than typical field measurements (mg/kg range). While such worst-case exposures are useful for hazard identification and mechanistic understanding, they may overestimate ecological risks when extrapolated to real soils. Therefore, future research should adopt tiered concentration designs that include environmentally realistic levels alongside higher doses, thereby bridging the gap between hazard assessment and actual risk quantification.

To comprehensively understand the changes in the crop rhizosphere microenvironment mediated by MPs, we propose the following suggestions to advance knowledge in this field. (1) Existing research on the toxic effects of MPs on plants has primarily focused on seed germination and plant biomass, whereas studies examining quality changes in various plant types—such as grain crops, cash crops, forage and green manure crops, and medicinal plants—remain relatively limited. Future efforts should therefore strengthen investigations into the effects of MPs on the safety and quality of the edible parts of plants. (2) Most studies on rhizosphere soil fauna have been conducted under laboratory conditions, which may not fully reflect the complexity of field soil environments, and the underlying mechanisms remain poorly understood. Hence, future research should emphasize field experiments to better elucidate the mechanisms by which soil fauna activities mitigate soil MP pollution, thereby providing a theoretical basis for reducing the harm of MPs to crops. (3) To further elucidate the regulatory role of viruses in carbon metabolism within MP-contaminated soil systems, it is necessary to establish methods for the isolation and identification of viral communities on MP surfaces, analyze their host specificity and auxiliary metabolic gene functions, and develop virus–microbe interaction models. Such approaches will help clarify the pathways through which MPs regulate carbon cycling by altering the ecological functions of viruses. (4) Relatively little attention has been paid to the effects and mechanisms of flame retardants on plants, and the impact mechanisms of degradation products such as plasticizers on plants remain unclear. Further exploration in this regard is warranted. (5) The molecular mechanisms underlying the responses of different plant species to MP stress are diverse and complex. The application of transcriptomics and metabolomics for single or integrated analyses still has certain limitations. Future studies could combine these with other techniques such as genomics and proteomics to further investigate the molecular response mechanisms of plants to MP stress.

## Figures and Tables

**Table 1:** The effect of MPs on microbial functional genes in soil.

Polymer Type	Dose (%)	Soil Type	Exposure Time/d	Reported Effects	Reference
PLA and PBAT	0.02	Fluvic Cambisol	120	BMPs significantly increased the abundance of genes related to CH_4_ production, especially genes related to the CO_2_ reduction pathway (such as fwdG, ftr, mch, and mtrH)	[90]
PLA, PHA, PBS and PBAT	0.5	Eutric Cambisol	730	BMPs treatment increased the expression of genes related to complex carbohydrate metabolism, such as SGA1, CELB, chba, bglX, abnA, as well as gmuG, manZ, manA, etc.	[91]
PBAT	1.0	Not provided	120	The addition of PBAT promotes an increase in the content of carbon degradation functional genes such as abfA, xylA, manB, mnp, glx, lig, and chiA.	[92]
PET	0.5	Hydragric Anthrosol	104	pmoA (+), pmoB (+), mcrA (−), & mcrB (−).	[93]
PE and PAN	0.5	Hydragric Anthrosol	104	mcrA (+) & pmoA (−).	[94]
PBAT	0.1, 0.5, 2.5	Rhodic Ferralsol	28	PBAT treatment increases the expression levels of genes related to the Calvin cycle, affecting the carbon fixation pathway	[95]
PE	1.0	Haplic Luvisol	45	Functional genes involved in tricarboxylic acid cycling (+).	[96]
PA	0.3–1.0	Hydragric Anthrosol	20	0.3% PA: mnp (+), chiA (+), accA (+), pccA (+), mcrA (+), & pmoA (+); 1% PA: mnp (−), chiA (−), mcrA (−), pmoA (−), mmoX (−), & pccA (+).	[97]
PLA	0.5, 1, 5	Eutric Cambisol	60	PLA can promote the enrichment of genes related to carbon conversion functions, such as sga, pgu, cex, exo-chi, abfA, xylA, and glx.	[51]
PLA	0.25, 2, 7	Not provided	35	PLA treatment increased the gene copy number of cbbL, a functional gene related to carbon fixation and photosynthesis.	[98]
PLA	1.0, 5.0	Luvisols	60	The enhancing effects of PLA on carbon cycle genes cbbL, cbbi, abfA, and Lac reached 37% to 200%, 46% to 2600%, 8% to 69%, and 91% to 600%, respectively.	[99]
PE	1.0	Hydragric Anthrosol	28	abfA (+) & mnp (+).	[81]
LDPE	0.01–1.0	Haplic Phaeozem	30	Virgin MPs: abfA (−) & manB (−); Aged MPs: sga (−), abfA (−), manB (−), xylA (−), & cex (−).	[80]
LDPE	0.5–1.5	Hydragric Anthrosol	41	The abundances of pmoA, pmoB, pmoC, mcrA, mcrB, mcrC, mcrD, and mcrG (varied with MP dose).	[87]

Note: “+” and “−” represent that the functional genes are up-regulated and down-regulated after adding MPs, respectively.

## Data Availability

No new data were created or analyzed in this study. Data sharing is not applicable to this article.

## Abbreviations

MPs: Microplastics; C: carbon; N: nitrogen; P: phosphorus; CO_2_: carbon dioxide; CH_4_: methane; PA: polyamide; PC: polycarbonate; PET: polyethylene terephthalate; PVC: polyvinyl chloride; PP: polypropylene; PMMA: polymethyl methacrylate; PS: polystyrene; LDPE: low-density polyethylene; HDPE: high-density polyethylene; PPA: Pipemidic Acid; PLA: Polylactic Acid; PBAT: Polybutylene terephthalate; PHBV: Poly(hydroxybutyrate-co-hydroxyvalerate); PBS: Poly(1,4-butanediol succinate); PU: Polyurethane; PHB: Polyhydroxybutyrate; PES: Polyethersulfone; PAN: Polyacrylonitrile; PTFE: Polytetrafluoroethylene.
